# Low Free Testosterone Is Independently Associated With Long‐Term Mortality in Men With Chronic Spinal Cord Injury

**DOI:** 10.1111/andr.70251

**Published:** 2026-05-01

**Authors:** D. Tienforti, L. Spagnolo, C. Moretto, G. Terrana, R. Di Geronimo, E. Perfetto, G. Felzani, M. G. Baroni, A. Barbonetti

**Affiliations:** ^1^ Andrology Unit Department of Clinical Medicine Life, Health and Environmental Sciences University of L'Aquila L'Aquila Italy; ^2^ Spinal Unit San Raffaele Sulmona Institute Sulmona Italy; ^3^ Neuroendocrinology and Metabolic Diseases IRCCS Neuromed Pozzilli Italy

**Keywords:** all‐cause mortality, androgen deficiency, comorbidity, hypogonadism, spinal cord injury, testosterone

## Abstract

**Background:**

Testosterone deficiency is highly prevalent in men with chronic spinal cord injury (SCI) and is associated with obesity, sarcopenia, systemic inflammation, and metabolic dysfunction. However, the independent prognostic role of low testosterone in long‐term mortality in this population remains unclear.

**Objectives:**

To investigate whether baseline calculated free testosterone (cFT) independently predicts all‐cause mortality in men with chronic SCI.

**Materials and Methods:**

We conducted a retrospective cohort study including 152 men with chronic SCI admitted to a rehabilitation center between 2017 and 2023. Participants were followed until death or December 2024 (median follow‐up 62 months). Severe hypogonadism was defined according to European Male Ageing Study criteria. Cox proportional hazards models were used to assess the association between testosterone and mortality. Fully adjusted models included age, comorbidities, inflammatory status, functional independence, injury duration, age at injury, autonomic dysreflexia, body mass index (BMI), HDL, and testosterone‐binding factors, including albumin. Prespecified parsimonious models (age + CCI or albumin) were also tested. Kaplan–Meier curves were compared by log‐rank test and model discrimination was evaluated using Harrell's C‐index.

**Results:**

Twenty‐four deaths (15.8%) occurred. Deceased patients had significantly lower cFT (48.9 vs. 85.4 pg/mL; *p* < 0.001) and lower serum albumin levels. In fully adjusted Cox models, lower cFT independently predicted mortality (HR 0.97 per pg/mL increase; 95% CI 0.95–0.98; *p* = 0.0139). Serum albumin was also strongly associated with mortality in multivariable models, confirming its role as a marker of nutritional and clinical frailty. The association between cFT and mortality remained robust in parsimonious models (HR 0.82 per 10 pg/mL increase; *p* = 0.001), including those adjusted for albumin. The age–cFT–CCI model showed good discrimination (Harrell's C‐index 0.85). ROC analysis identified an internally derived threshold of 59.55 pg/mL (AUC 0.839), with significantly lower survival in men below this threshold (log‐rank *p* < 0.0001).

**Discussion and Conclusion:**

Low cFT independently predicts all‐cause mortality in men with chronic SCI. These findings suggest that cFT may represent an integrative biomarker of systemic frailty beyond markers of nutritional and clinical status in this high‐risk population and warrant confirmation in prospective multicenter studies.

## 1 Introduction

Spinal cord injury (SCI) is a chronic and life‐altering condition associated with profound metabolic, endocrine, and cardiovascular dysregulation, significantly impacting long‐term survival. Advances in acute care and rehabilitation medicine have markedly improved life expectancy in individuals with chronic SCI [1]. however, premature mortality remains a major concern, with cardiovascular diseases (CVD), metabolic disorders, and endocrine dysfunction being the leading causes of death [2, 3].

Metabolic alterations are central to this excess mortality. SCI is characterized by reduced lean mass, increased visceral adiposity, insulin resistance, dyslipidemia, and chronic low‐grade inflammation, which collectively predispose to metabolic syndrome, type 2 diabetes, and CVD [4, 5]. Neurogenic obesity, with its distinct phenotype of fat redistribution, muscle atrophy, and sedentary lifestyle, further differentiates SCI from obesity in the general population [6].

Among the endocrine disturbances accompanying this metabolic derangement, testosterone (T) deficiency is one of the most prevalent and underrecognized abnormalities. Up to 50% of men with SCI exhibit low T levels, particularly those with complete motor loss or higher lesion levels [7, 8, 9]. The underlying mechanisms are multifactorial, involving hypothalamic–pituitary axis suppression, visceral adiposity, chronic systemic inflammation, and impaired neuromodulatory control of gonadotropin secretion [8, 10].

In the general population, low T is a well‐established risk factor for adverse cardiometabolic outcomes. T exerts anabolic effects on muscle, promotes insulin sensitivity, and modulates lipid metabolism; its deficiency is associated with sarcopenia, increased visceral fat, insulin resistance, and chronic low‐grade inflammation, all of which contribute to CVD risk [11, 12, 13, 14]. Large prospective cohorts, including the European Prospective Investigation into Cancer and Nutrition (EPIC)‐Norfolk and Osteoporotic Fractures in Men Study (MrOS), have demonstrated inverse associations between T levels and all‐cause and cardiovascular mortality, particularly in aging men and those with chronic diseases [15, 16, 17].

Whether these findings translate to SCI remains unknown. Despite the high prevalence of hypogonadism and its strong pathophysiological link to the neurogenic obesity and metabolic dysfunction characteristic of SCI, no study has specifically evaluated the prognostic significance of T levels in this population. Importantly, prior research suggests that the association between T deficiency and mortality is strongest in severe hypogonadism [18, 19], whereas borderline reductions in T may largely reflect chronic illness or frailty rather than a direct causal pathway.

This study addresses this gap by investigating the association between baseline T levels and long‐term all‐cause mortality in men with chronic SCI. To improve clinical relevance and reduce potential misclassification, we focused on severe hypogonadism, which is more consistently associated with adverse outcomes. By examining whether androgen deficiency independently predicts survival, we aim to clarify its prognostic significance and its potential value as an integrative marker of systemic frailty, with implications for metabolic and nutritional monitoring and for tailoring rehabilitation programs in this high‐risk population.

## 2 Patients and Methods

### 2.1 Participants

This retrospective cohort study included 152 adult men with chronic traumatic SCI who underwent a rehabilitation cycle at the San Raffaele Rehabilitation Center (Sulmona, Italy) between January 2017 and December 2023. Inclusion criteria were traumatic SCI of at least one‐year's duration, neurologically stable condition, absence of acute medical conditions interfering with or contraindicating rehabilitation, age ≥18 years, and no androgen replacement therapy at admission.

### 2.2 Study Design

Upon admission, participants underwent a comprehensive medical history assessment, physical and neurological examination, and laboratory testing. Chronic comorbidities were recorded and quantified using the Charlson Comorbidity Index (CCI), calculated via an online tool (https://www.mdcalc.com/calc/3917/charlson‐comorbidity‐index‐cci), which is a validated predictor of survival [20]. Patients were followed from enrollment until death or the end of follow‐up (December 2024). No participants initiateds T replacement therapy during the follow‐up period. The study was approved by the Ethics Committee of the Provinces of L'Aquila and Teramo, Italy (Approval Code: 11/CE/15, May 7, 2015), and was conducted in accordance with the principles of the Declaration of Helsinki. All participants provided written informed consent for the use of their clinical data for research purposes.

### 2.3 Clinical Assessments

Neurological status was evaluated according to the International Standards for Neurological Classification of Spinal Cord Injury (ISNCSCI) and graded using the American Spinal Injury Association (ASIA) Impairment Scale (AIS), which classifies the severity and completeness of neurological impairment [21]. Pain intensity was assessed using the numerical rating scale (NRS) [22], and functional independence in activities of daily living (ADL) was evaluated with the Spinal Cord Independence Measure (SCIM), a validated 19‐item scale reflecting global functional autonomy and widely used as a clinical outcome measure in SCI [23].

### 2.4 Anthropometric and Blood Pressure Measurements

Body weight was measured using a professional wheelchair scale (Wunder SA BI Srl, Monza, Italy). Height was estimated with a segmented anthropometric approach, with patients in a supine position, legs extended, feet in dorsiflexion, and head aligned with the Frankfurt plane. Body mass index (BMI) was calculated using standard methods as weight (kg) divided by height squared (m^2^). Systolic and diastolic blood pressure were recorded as the mean of three consecutive morning measurements during the first 3 days of hospitalization.

### 2.5 Laboratory Assessments

Fasting venous blood samples were collected between 8:00 and 9:00 AM on the morning following admission, after at least 12 h of fasting. Total testosterone (TT) was measured using chemiluminescent immunoassay (Ortho‐Clinical Diagnostics, Johnson & Johnson, New Brunswick, NJ, USA), while sex hormone‐binding globulin (SHBG) and serum albumin were measured using chemiluminescent immunoassay (Medical Systems, Genoa, Italy). TT levels were measured at baseline, at the time of admission to the rehabilitation program, when participants were clinically stable and free from acute conditions. Calculated free testosterone (cFT) was derived from TT, SHBG, and albumin concentrations using the Vermeulen equation [24], a widely validated method for estimating free T in clinical and research settings. TT, SHBG, and albumin were all measured in the same fasting morning blood sample collected between 08:00 and 09:00 AM. T levels were measured once at baseline and were not confirmed by repeat sampling. cFT was calculated using the International Society for the Study of the Aging Male (ISSAM) online calculator (http://www.issam.ch/freetesto.htm), based on the law‐of‐mass‐action equation originally described by Vermeulen et al. [24]. According to the European Male Ageing Study (EMAS) criteria [25], androgen deficiency was classified as mild (TT <3.2 ng/mL) or severe (concomitant presence of TT <3.2 ng/mL and cFT <64 pg/mL). Given the ongoing debate on the clinical significance of mild reductions in T, analyses focused on severe androgen deficiency, which is more consistently associated with adverse outcomes [18, 19]. Other biochemical and hematological parameters were measured using standard commercial kits (Instrumentation Laboratory Company, Lexington, MA, USA).

### 2.6 Metabolic and Inflammatory Markers

Insulin resistance was estimated using the Homeostatic Model Assessment for Insulin Resistance (HOMA‐IR) formula [26]. Low‐density lipoprotein (LDL) cholesterol was calculated using the Friedewald formula when applicable [27]. Systemic inflammation was defined as C‐reactive protein (CRP) >5 mg/L and/or erythrocyte sedimentation rate (ESR) >15 mm/h.

### 2.7 Statistical Analysis

Statistical analyses were performed using R software (version 4.5.1, 2025; The R Foundation for Statistical Computing, Vienna, Austria). Data distribution was assessed using the Shapiro–Wilk test. Continuous variables were compared between survivors and deceased patients using the Wilcoxon rank‐sum test, while categorical variables were compared using the chi‐square test or Fisher's exact test, as appropriate.

To investigate the association between T levels and all‐cause mortality, both TT and cFT were initially analyzed as continuous variables. Multivariable Cox proportional hazards regression models were fitted to assess their independent predictive value, with sequential adjustment for age, comorbidity burden (Charlson comorbidity index [CCI]), inflammatory status, functional independence (SCIM score), injury duration, age at injury, autonomic dysreflexia, BMI, high‐density lipoprotein (HDL) cholesterol, SHBG, and albumin, based on clinical relevance. Given the relatively limited number of mortality events (*n* = 24), particular attention was paid to the risk of model overfitting. Covariates were grouped into clinically coherent macro‐concepts representing major domains potentially confounding the association between T levels and mortality, including age/comorbidity burden, nutritional and functional status, metabolic profile, inflammatory status, and protein‐binding factors (Table S1). The proportional hazards assumption was tested using time‐dependent interaction terms.

Given the limited number of deaths and the potential influence of nutritional and clinical frailty on both circulating cFT and mortality risk, we additionally conducted a set of prespecified parsimonious Cox proportional hazards models to assess the robustness of the association between cFT and all‐cause mortality while minimizing the risk of model overfitting. These models included cFT and age, with the addition of one clinically relevant covariate at a time, selected a priori to represent key confounding domains, namely nutritional and functional status (serum albumin) or comorbidity burden (CCI). In these analyses, hazard ratios were expressed per 10 pg/mL increase in cFT. Model discrimination was evaluated using Harrell's C‐index in a prespecified parsimonious model including age, cFT, and CCI.

Receiver operating characteristic (ROC) curve analysis was used to evaluate the discriminatory ability of baseline cFT for all‐cause mortality and to derive an internally defined risk stratification threshold based on the Youden index. Sensitivity, specificity, and the area under the curve (AUC) were reported. Internal validity of the ROC‐based threshold was assessed using bootstrap resampling (2000 iterations), with estimation of the AUC and Youden‐index optimal cut‐off in each resample, as well as calculation of an optimism‐corrected AUC.

Kaplan–Meier survival curves were generated to compare survival between groups dichotomized according to the ROC‐derived cFT threshold, and differences were assessed using the log‐rank test.

As a sensitivity analysis, cFT values were further categorized into quartiles, and mortality rates were compared across strata to evaluate risk gradients across the distribution of cFT based on the observed values in the cohort. Graphical outputs of this analysis are provided in the Supporting Information. All statistical tests were two‐sided, and a *p*‐value < 0.05 was considered statistically significant.

## 3 Results

During the observation period, 24 deaths were recorded, accounting for 15.8% of the study population. As summarized in Table 1, deceased patients were older, had a longer duration of SCI, a higher comorbidity burden, and greater BMI compared with survivors. Systemic inflammation and autonomic dysreflexia were also more prevalent among deceased individuals, who additionally showed lower levels of functional independence in ADL.

**TABLE 1:** Baseline characteristics of the study population.

	Survivors (*n* = 128)	Died (*n* = 24)	*p* value
**Physiological and life‐style variables**			
Age (years)	54.2 ± 12.1	63.8 ± 11.6	< 0.001
Current smokers, *n* (%)	52 (41)	12 (50)	0.57
Alcohol (≥1 drink/day), *n* (%)	53 (42)	12 (50)	0.62
**Clinical and injury‐related variables**			
Severe androgen deficiency, *n* (%)	25 (19.5)	20 (83.0)	< 0.001
BMI (kg/m^2^)	26.1 ± 3.4	28.7 ± 4.1	0.03
Systolic blood pressure (mmHg)	113.9 ± 13.8	110.7 ± 13.4	0.32
Diastolic blood pressure (mmHg)	71.7 ± 7.9	70.2 ± 9.4	0.41
Age at injury (years)	39.6 ± 18.7	55.7 ± 15.2	<0.001
DOI (months)	180 (95–240)	220 (150–300)	0.02
CCI	3 (1–5)	5 (3–7)	0.01
Pressure sores, *n* (%)	23 (18)	8 (35)	0.13
Autonomic dysreflexia, *n* (%)	12 (9.4%)	8 (33.3%)	0.001
Functional independence (SCIM score)	51.1 ± 21.3	36.8 ± 19.1	0.003
Numeral Rating Scale (NRS) pain score	3.6 ± 2.8	3.7 ± 2.5	0.99
**Neurological level of the lesion**			
Cervical level, *n* (%)	45 (37)	11 (46)	0.53
Thoracic‐lumbar level, *n* (%)	78 (63)	13 (54)	0.53
**Lesion completeness**			
Complete motor lesion (ASIA A‐B), *n* (%)	76 (60)	13 (54)	0.73
Incomplete motor lesion (ASIA C‐D), *n* (%)	50 (40)	11 (46)	0.73
**Blood biometric measures**			
TT (ng/mL)	4.1 ± 1.9	2.1 ± 1.1	<0.001
cFT (pg/mL)	85.4 ± 21.2	48.9 ± 17.6	<0.001
SHBG (nmol/L)	26 ± 13.8	37.5 ± 22.6	0.001
Albumin (g/dL)	4.1 ± 0.5	3.4 ± 0.6	<0.001
Hemoglobin (g/dL)	13.8 ± 1.4	12.3 ± 1.4	<0.001
Hematocrit (%)	41.3 ± 5.3	37.6 ± 3.8	0.001
HOMA‐IR	2 ± 1.8	2.8 ± 3.2	0.15
Total cholesterol (mg/dL)	173.3 ± 38.7	161.3 ± 45.9	0.19
HDL (mg/dL)	48.3 ± 11.5	39.2 ± 9.8	0.004
LDL (mg/dL)	106.2 ± 31.8	98.5 ± 37.8	0.33
Triglycerides (mg/dL)	130.3 ± 64.7	142 ± 77.5	0.44
AST (U/L)	19.8 ± 17.8	21.8 ± 10.7	0.60
ALT (U/l)	25.9 ± 39.9	19.9 ± 10.6	0.47
Creatinine (mg/dL)	0.8 ± 0.2	0.8 ± 0.3	0.67
Azotemia (mg/dL)	37.1 ± 12.6	39.6 ± 13.7	0.40
Albumin‐corrected calcium (mg/dL)	9.2 ± 0.6	9.2 ± 0.7	0.27
Sodium (mmol/L)	140.4 ± 2.6	140.6 ± 1.7	0.77
Potassium (mmol/L)	4.2 ± 0.3	4.2 ± 0.3	0.64
Systemic inflammation, *n* (%)	18 (14.1%)	10 (41.7%)	<0.001

*Note*: Data are presented as mean ± SD or median (IQR), unless otherwise specified. Percentages are calculated on available data.

Abbreviations: ALT, alanine aminotransferase; ASIA, American Spinal Injury Association;AST, aspartate aminotransferase; BMI, body mass index; CCI, Charlson comorbidity index; cFT, calculated free testosterone; DOI, duration of injury; HDL, high‐density lipoprotein; HOMA‐IR, homeostatic model assessment for insulin resistance; LDL, low‐density lipoprotein; SCIM, spinal cord independence measure; SHBG, sex hormone‐binding globulin; TT, total testosterone. Severe androgen deficiency was defined as concomitant presence of TT <3.2 ng/mL and cFT <64 pg/mL. Systemic inflammation was defined as C‐reactive protein (CRP) levels >5 mg/L and/or erythrocyte sedimentation rate (ESR) >15 mm/h.

Regarding biochemical parameters, deceased individuals exhibited markedly lower cFT levels (48.9 vs. 85.4 pg/mL, *p* < 0.001), as well as lower serum albumin and HDL cholesterol concentrations (Table 1). Severe androgen deficiency was significantly more prevalent among deceased patients than among survivors (83.3% vs. 19.5%, *p* < 0.001).

### 3.1 Association between Calculated Free Testosterone and Mortality

In multivariable Cox regression analyses, both TT and cFT were inversely associated with all‐cause mortality, although cFT emerged as the more robust and consistent predictor across models (Table 2). In the crude model (Model I), lower TT was strongly associated with increased mortality risk (Hazard Ratio [HR] 0.45; 95% CI 0.29–0.68; *p* = 0.0002). This association remained significant after adjusting for age (Model II: HR 0.39; 95% CI 0.23–0.66; *p* = 0.0005) and additional clinical covariates including comorbidities, inflammation, injury duration, and SCIM score (Model III: HR 0.32; 95% CI 0.17–0.63; *p* = 0.0008). However, in the fully adjusted model (Model IV), which included BMI, HDL cholesterol, SHBG, and serum albumin, the association between TT and mortality lost statistical significance (HR 0.48; 95% CI 0.23–1.01; *p* = 0.0542), suggesting possible collinearity with SHBG and albumin.

**TABLE 2:** Association of total testosterone and calculated free testosterone with all‐cause mortality: Multivariable Cox proportional hazards models.

	Mortality							
	Model I		Model II		Model III		Model IV	
	HR (95%CI)	*p* value	HR (95%CI)	*p* value	HR (95%CI)	*p* value	HR (95%CI)	*p* value
TT	0.45 (0.29–0.68)	**0.0002**	0.39 (0.23–0.66)	**0.0005**	0.32 (0.17–0.63)	**0.0008**	0.48 (0.23–1.01)	0.0542
cFT	0.96 (0.95–0.98)	**<0.0001**	0.97 (0.95–0.98)	**0.0005**	0.97 (0.94–0.99)	**0.0027**	0.97 (0.95–0.98)	**0.0139**

*Note*: Values are expressed as hazard ratio (HR) per unit increase of TT (ng/mL) and cFT (pg/mL) with 95% confidence interval (CI).

Model I = unadjusted; Model II = adjusted for age; Model III = Model II + comorbidity, inflammation, SCIM score, injury duration, age at injury, and autonomic dysreflexia; Model IV = Model III + BMI, HDL (with SHBG and albumin for TT).

Abbreviations: BMI, body mass index; cFT, calculated free testosterone; HDL, high density lipoprotein; SCIM, Spinal Cord Independence Measure; SHBG, sex hormone binding globulin; TT, total testosterone.

In contrast, lower cFT levels consistently predicted higher mortality risk in all models. In the unadjusted model (Model I), the HR per 1 pg/mL increase in cFT was 0.96 (95% CI 0.95–0.98; *p* < 0.0001). This association remained significant after adjusting for age (Model II: HR 0.97; 95% CI 0.95–0.98; *p* = 0.0005) and for a broader set of clinical variables (Model III: HR 0.97; 95% CI 0.94–0.99; *p* = 0.0027). Importantly, in the fully adjusted model (Model IV), cFT remained an independent predictor of mortality (HR 0.97; 95% CI 0.95–0.98; *p* = 0.0139), indicating a stable association even after accounting for demographic, metabolic, and T‐binding factors.

Taken together, these findings indicate a significant inverse association between T levels and mortality in men with chronic SCI, with cFT showing a more consistent association than TT across multivariable‐adjusted models.

To further assess the robustness of the association between cFT and mortality and to minimize the risk of overfitting, we performed prespecified parsimonious Cox regression models including only age and one additional clinically relevant covariate. In these clinically more interpretable models, cFT remained independently associated with all‐cause mortality after adjustment for age alone (HR per 10 pg/mL increase: 0.81, 95% CI 0.72–0.92), as well as after additional adjustment for albumin or CCI (Table 3). The prespecified parsimonious model including age, cFT, and CCI showed good discrimination for all‐cause mortality (Harrell's C‐index = 0.85).

**TABLE 3:** Parsimonious Cox proportional hazards models for the association between calculated free testosterone and all‐cause mortality.

Model	Covariates	HR per 10 pg/mL cFT (95%CI)	*p*
P1	Age	0.81 (0.72‐0.92)	0.001
P2	Age + Albumin	0.82 (0.72‐0.92)	0.001
P3	Age + CCI	0.82 (0.72‐0.93)	0.001

*Note*: Parsimonious Cox proportional hazards regression models evaluating the association between baseline calculated free testosterone (cFT) and all‐cause mortality in men with chronic spinal cord injury. Hazard ratios (HRs) are expressed per 10 pg/mL increase in cFT. Model P1 was adjusted for age only. Model P2 was adjusted for age and serum albumin. Model P3 was adjusted for age and Charlson comorbidity index (CCI). These models were prespecified to assess the robustness of the association while minimizing the risk of overfitting due to the limited number of events. All models met the proportional hazards assumption.

Consistent with the role of nutritional and clinical frailty, in additional analyses, serum albumin was evaluated as a candidate predictor of all‐cause mortality. Lower albumin levels were significantly associated with increased mortality risk in unadjusted (HR 0.12, 95% CI 0.05–0.31; *p* < 0.0001) and age‐adjusted models (HR 0.25, 95% CI 0.09–0.70; *p* = 0.0088) and remained significant in the broader multivariable model including age, CCI, inflammatory status, SCIM score, injury duration, age at injury, autonomic dysreflexia, BMI, and HDL cholesterol, (HR 0.046, 95% CI 0.008–0.259; *p* = 0.0005). However, in an additional model including age, albumin, and cFT, both variables remained independently associated with mortality (cFT: HR 0.980, 95% CI 0.968–0.992; *p* = 0.0013; albumin: HR 0.237, 95% CI 0.086–0.649; *p* = 0.0051), supporting the independent prognostic role of cFT beyond nutritional status.

In additional exploratory analyses, bioavailable T was also inversely associated with mortality after adjustment for age (HR 0.72, 95% CI 0.61–0.86; *p* = 0.0002), confirming a consistent relationship between androgen status and survival.

### 3.2 Discriminatory Power of Calculated Free Testosterone for Mortality Risk

**FIGURE 1:**
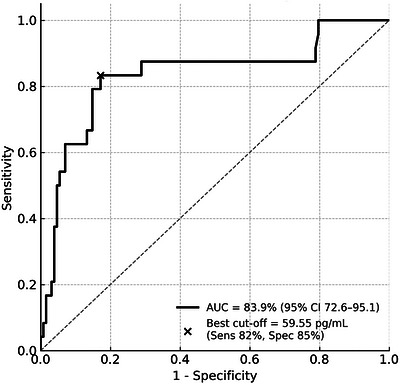
Receiver operating characteristic (ROC) curve of calculated free testosterone for all‐cause mortality. ROC curve illustrating the discriminative ability of baseline calculated free testosterone for all‐cause mortality in men with chronic spinal cord injury. The area under the curve (AUC) was 0.839 (95% CI 0.726–0.951). The cross marks the internally derived optimal cut‐off value of 59.55 pg/mL, identified by the Youden index, corresponding to a sensitivity of 82% and a specificity of 85%.

ROC curve analysis identified a cFT threshold of 59.55 pg/mL, below which the risk of mortality significantly increased. This cut‐off, which was met by 27.6% of the study population, yielded a sensitivity of 82% and specificity of 85% for predicting all‐cause mortality, with an AUC of 0.839 (95% CI, 0.726–0.951) (Figure 1).

Internal bootstrap validation (2000 resamples) supported the stability of discrimination (median AUC 0.84; 95% bootstrap CI 0.72–0.93; optimism‐corrected AUC 0.83). The Youden‐derived cut‐off showed greater sampling variability (median 59.3 pg/mL; 95% bootstrap CI 37.0–70.3 pg/mL). The identified threshold was used for risk stratification rather than for diagnostic or therapeutic decision‐making.

### 3.3 Survival Analysis Based on Calculated Free Testosterone Levels

**FIGURE 2:**
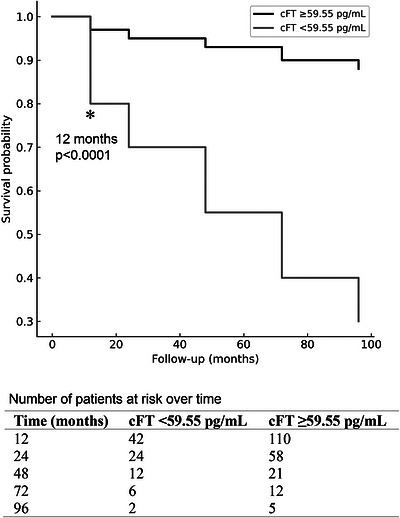
Kaplan–Meier survival curves stratified by calculated free testosterone levels. Kaplan–Meier survival curves for all‐cause mortality stratified according to calculated free testosterone (cFT) levels using the internally derived ROC‐based threshold of 59.55 pg/mL. Individuals with cFT <59.55 pg/mL showed significantly lower survival compared with those with cFT ≥59.55 pg/mL, with an early and sustained separation of the curves over follow‐up (log‐rank *p* < 0.0001). The adjacent table to the plot reports the number of patients at risk in each group at predefined follow‐up time points.

Survival analysis further corroborated the prognostic relevance of low cFT levels. Kaplan–Meier survival curves stratified by the ROC‐derived threshold of 59.55 pg/mL showed significantly reduced survival among individuals with cFT below this cut‐off, with an early and sustained separation of the curves evident from approximately 12 months of follow‐up (log‐rank *p* < 0.0001) (Figure 2).

### 3.4 Sensitivity Analyses

In sensitivity analyses, cFT was categorized into quartiles, revealing a marked concentration of mortality in the lowest quartile (Q1: ≤55.3 pg/mL; 50.0%), which differed significantly from all other quartiles, whereas mortality rates were comparable across Q2–Q4 (Figure S1). These findings further support the robustness of the ROC‐derived threshold.

## 4 Discussion

To our knowledge, this is the first study to specifically investigate the prognostic value of cFT in men with chronic, clinically stable SCI. We found that lower baseline cFT levels independently predicted higher all‐cause mortality over a follow‐up ranging from 12 to 96 months with a hazard ratio of 0.98 per pg/mL increase in cFT (95% CI 0.97–1.00). Notably, the association between cFT and mortality was consistent across fully adjusted and parsimonious models, supporting the robustness of the finding despite the limited number of events. When expressed on a clinically more meaningful scale, the association between cFT and mortality remained substantial, with an approximately 18% lower risk per 10 pg/mL increase. An internally derived ROC‐based threshold of 59.55 pg/mL was identified for risk stratification, below which mortality risk was substantially higher. Although slightly lower than the 64 pg/mL threshold proposed for the diagnosis of biochemical hypogonadism in the general population [28], nearly one‐third of our cohort fell below this value at baseline. The early separation of survival curves suggests that low cFT may capture an early vulnerability phenotype rather than a late‐stage risk marker.

Our sensitivity analyses with quartile‐based stratification confirmed the robustness of this finding: the ROC‐derived threshold was located close to the boundary between the first and second quartiles, reflecting the steep increase in mortality risk at low cFT values, while the lowest quartile identified the subgroup with the highest absolute risk. This converging evidence supports the biological plausibility and clinical relevance of the ROC‐derived cut‐off.

The relationship between T status and mortality has been examined in various forms of hypogonadism. In a large cohort of patients with Klinefelter syndrome, increased mortality was observed, although the relative contribution of hypogonadism versus chromosomal abnormality was unclear [29]. In hypopituitarism, higher mortality was reported in individuals with gonadotropin deficiency [30], though sex‐specific analyses were lacking.

In late‐onset hypogonadism (LOH), findings have been heterogeneous. The EPIC‐Norfolk study reported an inverse association between T and all‐cause, cardiovascular, and cancer mortality [15]. Similar associations were observed in the Rancho Bernardo [31] and SHIP [32] studies, whereas other cohorts, such as the Massachusetts Male Aging Study [33] and the Tromsø Study [34], reported weaker or non‐significant results. A meta‐analysis including nearly 300,000 men confirmed the association between low TT and both all‐cause and cardiovascular mortality [35], although heterogeneity and the observational design limit causal inference. More recently, a large systematic review and dose–response meta‐analysis of prospective cohort studies reported a significant association between TT and all‐cause mortality in men, whereas neither free nor bioavailable T showed a consistent association with mortality risk [36]. These findings, however, derive from general population cohorts and may not be directly applicable to patients with chronic SCI, who are characterized by profound alterations in body composition, inflammation, and protein‐binding dynamics that may modify the relationship between androgen status and clinical outcomes. In this setting, cFT may capture a disease‐specific vulnerability phenotype and therefore may have a different prognostic significance from that observed in community‐based populations.

Unlike age‐related LOH, SCI often results in early‐onset hypogonadism through multifactorial mechanisms [8, 37]. Bauman et al. demonstrated a higher prevalence of hypogonadism across all age decades in SCI men compared with population controls [38]. In our study, cFT ‐but not TT‐ remained an independent predictor of mortality in the fully adjusted model, underscoring the greater biological relevance of cFT, which accounts for albumin and SHBG concentrations [24].

Although bioavailable T can also be derived from TT, SHBG, and albumin, we focused on cFT as the primary exposure variable. cFT represents the unbound fraction of circulating T and is considered the biologically active component at the tissue level. Moreover, cFT calculated using the Vermeulen equation is a widely validated and standardized measure. In contrast, bioavailable T includes the albumin‐bound fraction and may be more influenced by variations in protein‐binding conditions, particularly in populations with altered nutritional and inflammatory status such as SCI. Consistent with this, in our exploratory analyses, bioavailable T showed a similar association with mortality, but did not provide additional conceptual or methodological advantages over cFT.

Deceased participants exhibited significantly lower cFT, higher BMI, and lower HDL cholesterol, but no differences in insulin sensitivity or blood pressure, likely reflecting the impact of autonomic dysregulation in SCI [39, 40]. Testosterone's anabolic effects on muscle are well documented [41, 42, 43], and deficiency may exacerbate sarcopenia, functional decline, and fracture risk [44]. Indirect markers of sarcopenia, hypoalbuminemia and reduced independence in ADL, were indeed more frequent among non‐survivors. Interestingly, serum albumin was also a strong predictor of mortality in our cohort, consistent with its established role as a marker of nutritional reserve and systemic frailty. However, the association between cFT and mortality persisted after adjustment for albumin, indicating that low cFT is unlikely to be merely a surrogate of poor nutritional status. Rather, albumin and cFT may capture partially distinct dimensions of vulnerability in men with chronic SCI.

Chronic low‐grade inflammation may also contribute to this association, as T downregulates pro‐inflammatory cytokines such as IL‐6 and TNFα [45, 46]. Although inflammatory markers were higher in non‐survivors, inflammation did not account for the cFT–mortality link in multivariable analysis. Additional mechanisms may include endothelial dysfunction [47, 48] and neuropsychological factors such as cognitive decline [49, 50], apathy [51, 52], and depression [53, 54], all previously associated with increased mortality risk [55, 56].

Strengths of this study include the use of centralized clinical and biochemical assessments, rigorous inclusion criteria, and the deliberate selection of a homogeneous cohort of men with chronic traumatic SCI in clinically stable condition, the most common profile encountered in specialized rehabilitation centers. This homogeneity minimizes clinical variability and enhances the interpretability of endocrine parameters in this high‐risk but understudied population [57]. In addition, none of the participants received T therapy during follow‐up, excluding potential confounding effects of exogenous androgen treatment on the observed associations. Additional strengths include the focus on cFT rather than TT alone, and the use of prespecified multivariable and parsimonious analytical approaches designed to balance confounding control with model robustness.

This study also has several limitations. First, the relatively small number of deaths (*n* = 24) inevitably limits statistical power and raises concerns about potential overfitting in fully adjusted Cox regression models. In survival analyses, statistical power is driven primarily by the number of events. Based on Schoenfeld's approximation and the observed variability of cFT in the cohort, the number of events in our study provided approximately 80% power to detect hazard ratios of about 0.79 or lower per 10 pg/mL increase in cFT (two‐sided alpha 0.05). Thus, the study was reasonably powered to detect associations of at least moderate magnitude, whereas smaller effects may have remained undetected. This should be considered when interpreting both the observed cFT association and the less robust findings for TT in the most adjusted models. To mitigate the issue of the small number of events, we adopted a modeling strategy based on predefined macroconcepts and complemented fully adjusted models with prespecified parsimonious analyses, which yielded consistent results. Importantly, the modest number of deaths also reflects the relatively young age of the cohort, while the observed 15.8% mortality rate over a median follow‐up of 62 months remains clinically meaningful in a chronic SCI population. Most covariates included in the final model cluster into a limited number of correlated clinical domains, namely age/comorbidity burden, nutritional and functional status, metabolic profile, inflammatory status, and androgen‐binding factors, thereby reducing the effective dimensionality of the model. Consistent with this, the association between low cFT and mortality was stable across all analytical approaches, including minimally adjusted and prespecified parsimonious models, as well as across multiple sensitivity analyses. Together, these considerations support the robustness of the observed association, although the findings should be regarded as hypothesis‐generating and warrant confirmation in larger prospective studies. Although model discrimination was good in parsimonious analyses, the limited number of events precluded a reliable assessment of model calibration; moreover, the fully adjusted models were designed for explanatory rather than predictive purposes. Given the limited number of events and the clinically stable chronic phase of the cohort at baseline, exclusion of early deaths to address potential reverse causation was not performed, as this would have substantially reduced statistical power. Similarly, non‐linear modeling approaches (e.g., restricted cubic splines) were not presented, as the limited number of events and the sparse distribution of observations at the extremes of cFT would have rendered such analyses unstable and potentially misleading due to overfitting and boundary effects. Additionally, the ROC‐derived cFT threshold should be interpreted as an internally derived, exploratory value for risk stratification within this cohort rather than as a clinically applicable cut‐off. Although discrimination remained stable after bootstrap validation, the relatively wide confidence interval around the Youden‐derived threshold reflects sampling variability and limits its immediate generalizability. Therefore, this cut‐off requires external validation in larger, independent cohorts before any potential clinical application. Whether identifying low cFT and intervening with T therapy improves hard outcomes in this population remains unknown and would require adequately powered randomized controlled trials. As another limitation, TT levels were assessed at baseline only, and longitudinal changes over time could not be evaluated. T levels were assessed at a single time point and were not confirmed by repeat measurements, as recommended for the clinical diagnosis of hypogonadism. However, given the prognostic nature of the study, baseline hormone levels were considered as exposure variables, in line with previous epidemiological investigations evaluating the association between T and mortality. Notably, TT measurements were obtained at baseline in clinically stable conditions and were not performed in temporal proximity to death, reducing the likelihood of reverse causation due to terminal illness. Furthermore, TT was measured using a chemiluminescent immunoassay rather than liquid chromatography–tandem mass spectrometry, which is currently considered the reference method. Nevertheless, the assay employed has demonstrated good agreement with LC–MS/MS within the concentration range observed in this cohort [58], and similar methodologies have been used in large prospective studies examining the association between T levels and mortality, including EPIC‐Norfolk and MrOS [15, 16]. In addition, detailed phenotyping was not available, including measures of body composition (e.g., DXA‐derived lean and fat mass), muscle strength, and patient‐reported outcomes such as quality of life, which could have helped clarify mechanistic links between androgen status, functional decline, and mortality risk. Finally, detailed and reliable cause‐specific mortality data were not available in this cohort. As this was a retrospective study, information on causes of death was derived from official death certificates, which frequently reported non‐specific entries (e.g., “cardio‐respiratory arrest”), precluding accurate classification of underlying causes. Therefore, formal analyses of cause‐specific mortality were not feasible. However, all‐cause mortality represents a robust and unbiased endpoint in prognostic studies, particularly in relatively small samples, and it avoids misclassification of causes of death. Accordingly, the pathophysiological considerations discussed should be interpreted as plausible mechanisms linking androgen deficiency to increased vulnerability and mortality risk, rather than as evidence of specific causal pathways.

In conclusion, low cFT (<59.55 pg/mL) independently predicts all‐cause mortality in men with chronic SCI. Regardless of causality, cFT appears to be a practical biomarker of clinical frailty in this population. From a clinical standpoint, these findings suggest that assessment of androgen status, preferably including cFT, may be considered in men with chronic SCI, particularly in those with features of systemic frailty or cardiometabolic risk. However, the present study does not support routine population‐wide screening, and the clinical utility of integrating cFT into standard follow‐up pathways, as well as the consistency of the observed association across specific SCI subgroups, require confirmation in larger multicenter prospective cohorts.

## Author Contributions

D.T. and A.B. originally conceived the study design, collected data, and performed statistical analysis. C.M., G.T., R.D.G., and E.P. contributed to statistical analysis. G.F contributed to data collection and critically revised the manuscript. M.G.B. contributed to statistical analysis and critically revised the manuscript. All authors approved the submitted final version of the manuscript.

## Funding

The authors have nothing to report.

## Conflicts of Interest

The authors declare no conflicts of interest.

## Supporting information




**Supporting File 1**: andr70251‐sup‐0001‐FigureS1.tif.

**Table S1**: Predefined macroconcepts and covariates included in the multivariable Cox proportional hazards models.

## Data Availability

The datasets generated and analyzed during the current study are available from the corresponding author upon reasonable request.
