# Supplementary figures and images for: Low Free Testosterone Is Independently Associated With Long‐Term Mortality in Men With Chronic Spinal Cord Injury
Source: Andrology. 2026 May 1;14(6):1712–22. doi: 10.1111/andr.70251 (PMC13432611; doi:10.1111/andr.70251)

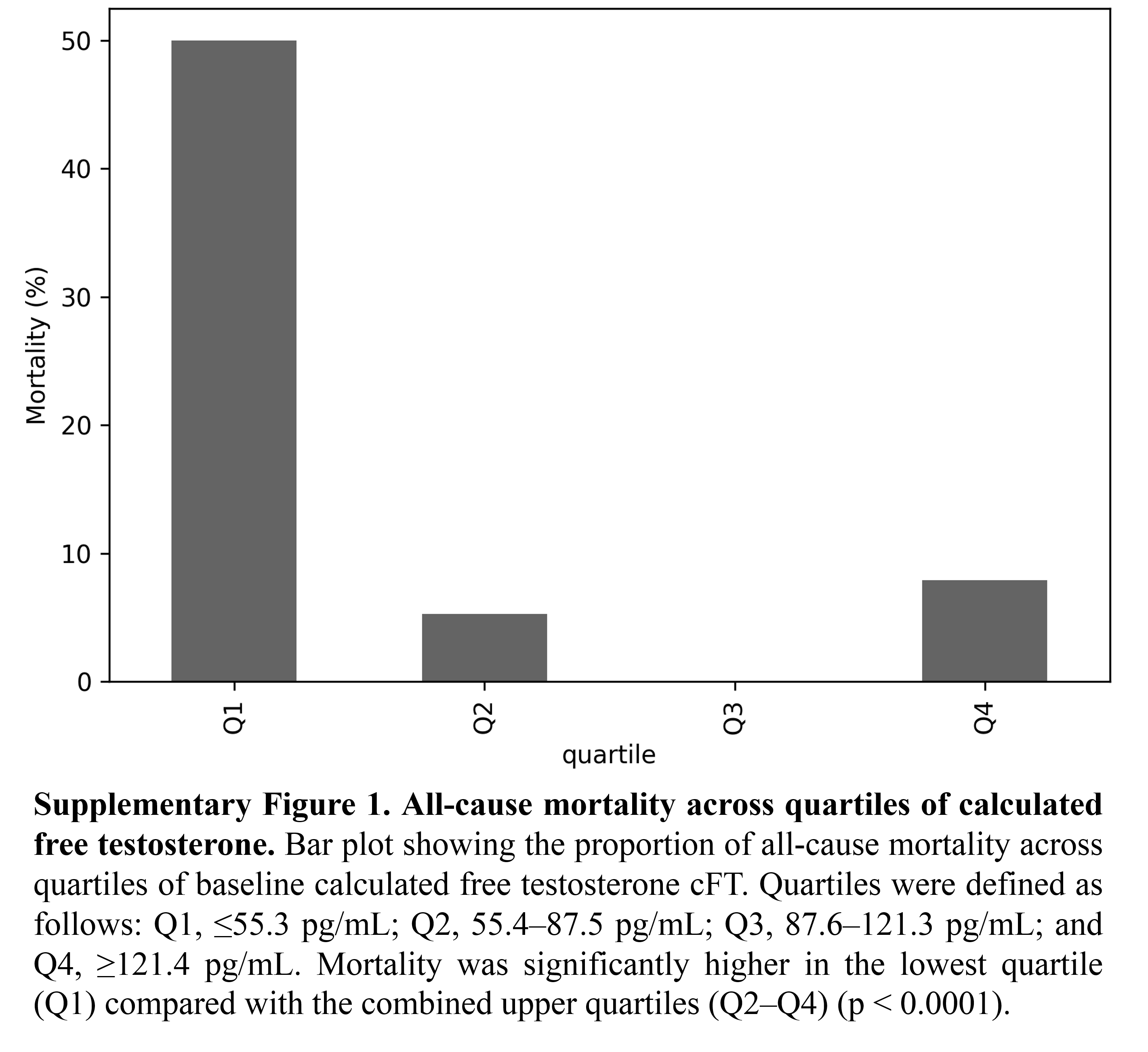

Supplement: Supplementary file 1 — Supporting File 1: andr70251‐sup‐0001‐FigureS1.tif. [file ANDR-14-1712-s001.tif]
