# Supplementary material for: Low Free Testosterone Is Independently Associated With Long‐Term Mortality in Men With Chronic Spinal Cord Injury
Source: Andrology. 2026 May 1;14(6):1712–22. doi: 10.1111/andr.70251 (PMC13432611; doi:10.1111/andr.70251)
Supplement: Supplementary file 2 — Table S1: Predefined macroconcepts and covariates included in the multivariable Cox proportional hazards models. [file ANDR-14-1712-s002.docx]

**Supplementary Table 1.** Predefined macro-concepts and covariates included in the multivariable Cox proportional hazards models.

| **Macro-concept** | **Covariates included in the models** | **Rationale for inclusion** |
| --- | --- | --- |
| Age / Comorbidity burden | Age; CCI; age at injury; duration of injury; autonomic dysreflexia | Control for baseline health status, disease severity, and chronicity of spinal cord injury |
| Nutritional and  functional status | Albumin; BMI; SCIM score | Capture nutritional reserves, sarcopenia risk, and overall functional capacity |
| Metabolic profile | HDL cholesterol | Account for lipid metabolism as a cardiovascular risk factor |
| Inflammatory status | Inflammatory status (CRP >5 mg/L and/or ESR >15 mm/h) | Adjust for chronic low-grade inflammation known to affect both testosterone and survival |
| Protein-binding factors | SHBG [only in TT models] | Adjust for testosterone bioavailability differences not explained by total testosterone alone |

Abbreviations: BMI, body mass index; CCI, Charlson Comorbidity Index; CRP, C-reactive protein; ESR, erythrocyte sedimentation rate; HDL, high-density lipoprotein; SCIM, Spinal Cord Independence Measure; SHBG, sex hormone-binding globulin; TT, total testosterone.
